# Fast selective edge-enhanced imaging with topological chiral lamellar superstructures

**DOI:** 10.1093/nsr/nwae247

**Published:** 2024-07-17

**Authors:** Wen Chen, Dong Zhu, Si-Jia Liu, Yi-Heng Zhang, Lin Zhu, Chao-Yi Li, Shi-Jun Ge, Peng Chen, Wan-Long Zhang, Xiao-Cong Yuan, Yan-Qing Lu

**Affiliations:** National Laboratory of Solid State Microstructures, Key Laboratory of Intelligent Optical Sensing and Manipulation, College of Engineering and Applied Sciences, and Collaborative Innovation Center of Advanced Microstructures, Nanjing University, Nanjing 210093, China; National Laboratory of Solid State Microstructures, Key Laboratory of Intelligent Optical Sensing and Manipulation, College of Engineering and Applied Sciences, and Collaborative Innovation Center of Advanced Microstructures, Nanjing University, Nanjing 210093, China; National Laboratory of Solid State Microstructures, Key Laboratory of Intelligent Optical Sensing and Manipulation, College of Engineering and Applied Sciences, and Collaborative Innovation Center of Advanced Microstructures, Nanjing University, Nanjing 210093, China; National Laboratory of Solid State Microstructures, Key Laboratory of Intelligent Optical Sensing and Manipulation, College of Engineering and Applied Sciences, and Collaborative Innovation Center of Advanced Microstructures, Nanjing University, Nanjing 210093, China; National Laboratory of Solid State Microstructures, Key Laboratory of Intelligent Optical Sensing and Manipulation, College of Engineering and Applied Sciences, and Collaborative Innovation Center of Advanced Microstructures, Nanjing University, Nanjing 210093, China; National Laboratory of Solid State Microstructures, Key Laboratory of Intelligent Optical Sensing and Manipulation, College of Engineering and Applied Sciences, and Collaborative Innovation Center of Advanced Microstructures, Nanjing University, Nanjing 210093, China; National Laboratory of Solid State Microstructures, Key Laboratory of Intelligent Optical Sensing and Manipulation, College of Engineering and Applied Sciences, and Collaborative Innovation Center of Advanced Microstructures, Nanjing University, Nanjing 210093, China; National Laboratory of Solid State Microstructures, Key Laboratory of Intelligent Optical Sensing and Manipulation, College of Engineering and Applied Sciences, and Collaborative Innovation Center of Advanced Microstructures, Nanjing University, Nanjing 210093, China; Nanophotonics Research Center, Institute of Microscale Optoelectronics, Shenzhen University, Shenzhen 518060, China; Nanophotonics Research Center, Institute of Microscale Optoelectronics, Shenzhen University, Shenzhen 518060, China; National Laboratory of Solid State Microstructures, Key Laboratory of Intelligent Optical Sensing and Manipulation, College of Engineering and Applied Sciences, and Collaborative Innovation Center of Advanced Microstructures, Nanjing University, Nanjing 210093, China

**Keywords:** edge detection, ferroelectric liquid crystal, topological structure, vector beam, fast response

## Abstract

Edge detection is a fundamental operation for feature extraction in image processing. The all-optical method has aroused growing interest owing to its ultra-fast speed, low energy consumption and parallel computation. However, current optical edge detection methods are generally limited to static devices and fixed functionality. Herein, we propose a fast-switchable scheme based on a ferroelectric liquid crystal topological structure. The self-assembled chiral lamellar superstructure, directed by the azimuthally variant photo-alignment agent, can be dynamically controlled by the polarity of the external electric field and respectively generates the vector beams with nearly orthogonal polarization distribution. Even after thousands of cycles, the horizontal and vertical edges of the object are selectively enhanced with an ultra-fast switching time of ∼57 μs. Broadband edge-enhanced imaging is efficiently demonstrated. This work extends the ingenious building of topological heliconical superstructures and offers an important glimpse into their potential in the emerging frontiers of optical computing for artificial intelligence.

## Teaser

Topological chiral lamellar superstructures enable dynamic light manipulation and fast selective edge-enhanced imaging in a wide band.

## INTRODUCTION

Image processing—the procedure of certain operations to extract practical information from images—plays a significant role in the rapid development of modern technology [1]. As an indispensable part, edge detection, which involves computing an image gradient to quantify the magnitude and direction of edges in an image, has considerable applications in medical imaging, face recognition and autonomous vehicles. Nowadays, facing the rapidly growing demands for fast computation, low energy consumption and parallel processing [2], all-optical computation is emerging as a promising avenue for edge detection [3]. To date, several optical differentiators have been proposed for real-time and efficient edge detection, including photonic crystals [4], photonic chips [5] and metasurfaces [6–13]. However, those devices and structures are usually fixed once fabricated and their functionality is always static. The highly limited non-reconfigurable photonic devices cast a shadow on the dynamic tunability of edge-enhanced imaging, thus dampening the potential of this technology.

Liquid crystals (LCs) are well known for their good sensitivity to external stimuli such as heat, electric/magnetic fields and light irradiation, and have undergone numerous developments in dynamic functional devices [14–17]. As an arresting LC material, ferroelectric chiral smectic C (SmC*) LC is characterized by the in-plane switching of LC directors under an external electric field and the corresponding ultra-fast electro-optical response [18–20]. Without any stimulation, ferroelectric liquid crystal (FLC) molecules rotate, surrounding the layer normal with a fixed tilt angle, resulting in the self-assembly of a helical lamellar structure. Three specific electro-optical modes have been discovered in FLCs, including the surface-stabilized FLC mode [21], deformed helix ferroelectric mode [22,23] and electrically suppressed helix (ESH) mode [24,25]. The ESH mode stands out due to its tunable optical axis with an ultra-high speed, where the chiral structure of FLCs is highly suppressed by a proper electric field. Successful attempts such as FLC wave plates [25], binary gratings [26] and Fresnel lens [27] have enriched the platform for active optics and photonics. If the reconfigurable optical axis is thoroughly investigated and the limitation of common binary FLC structures is resolved, then FLC can be rationally expected to be a strong candidate for the new scheme of dynamic edge-enhanced imaging.

In this work, we propose a fast selective and polychromatic optical edge detection strategy based on inhomogeneously nanostructured FLCs. The photo-alignment technology is utilized to build a chiral lamellar structure with azimuthal variation, constructing topological heliconical superstructures in FLCs. Due to the unique mechanism of the ESH mode, the overall space-variant optical axis rotates synchronously with opposite electric field polarities. Nearly orthogonal vector beams can be dynamically generated under the same incidence. Based on a conventional 4*f* system cooperated with crossed polarizers, real-time and high-quality edge-enhanced imaging can be realized. Horizontal or vertical edges can be selectively highlighted by alternating the polarity of the applied electric field. The measured switching time is as fast as 57 μs and the dynamic functionality remains great over 2000 cycles. Since unmodulated light is filtered by the crossed polarizers here, high-contrast edge imaging is also efficiently achieved throughout an ultra-broad spectrum. Our proposed strategy extends an innovative course for the self-assembled construction of hierarchical superstructures and inspires their applications in machine vision, recognition and sensing.

## RESULTS

### Principle of dynamic switchable edge-enhanced imaging

**Figure 1.**
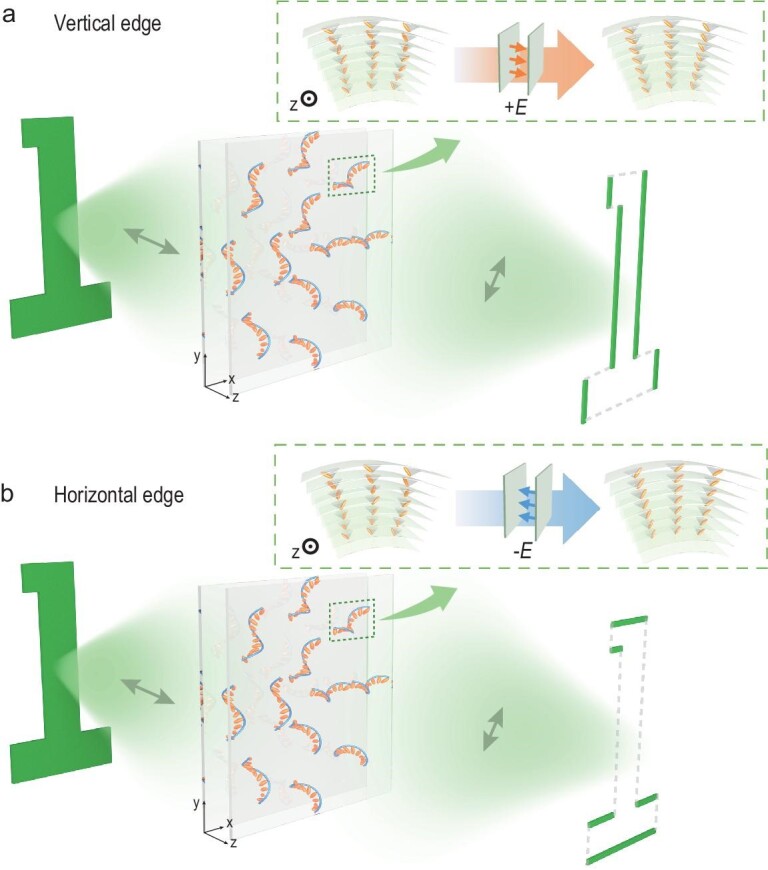
Schematic of the FLC topological superstructures for fast selective edge-enhanced imaging. The ellipsoid represents the unit director of the FLC molecules. The helical lines and the planes illustrate the chiral lamellar structure. The double-ended arrows designate the direction of the polarizer or analyser. (a) Vertical edge imaging under a positive electric field. Inset: the helical structure is suppressed and the FLC molecules rotate to the left side of the core parallel to the substrates. (b) Horizontal edge imaging under a negative electric field. Inset: the helical structure is suppressed and the FLC molecules rotate to the right side of the core parallel to the substrates.

In the SmC* LC phase, the equilibrium orientational structures of FLC molecules can be described as a helical twisting lamellar structure [28]. The FLC molecules self-assemble and align averagely on the cone with tilt angle *θ*, resulting in the formation of the FLC helix with pitch *P*_0_. The twisting axis is perpendicular to the smectic layers and the spontaneous polarization vector *P*_S_ spirals around the normal of the smectic layer, always directed perpendicular to it. Under planar boundary conditions, the FLC helix lies parallel to the substrates. In the ESH mode of FLCs [24], an electric field perpendicular to the helix axis completely suppresses the helix at *V* ≥ *V*_th_, where *V*_th_ is the threshold voltage. Application of the electric field causes FLC molecules to synchronously rotate along the surface of the helical cone towards a specific side parallel to the substrates, as demonstrated in the inset of Fig. 1a and b. The exact rotational direction (+*θ* or –*θ*) depends on the polarity of the applied electric field. The electric torque resulting from the interaction between the spontaneous polarization *P*_S_ of the FLCs and the external electric field switches the final director orientation from one side to the other. Thus, the equivalent local optical axis of the ESH–FLCs can be switched between +*θ* and –*θ* by changing the electric field polarity.

To achieve the desired optical differential operation for edge imaging, we introduce a delicate pattern with azimuthal variation into FLC hierarchical superstructures. The orientation angle of the equivalent optical axis varies azimuthally and a topological defect is formed in the central region. This similar structure is also known as a ‘*q*-plate’ [29,30], with the equivalent optical axis of such an inhomogeneous half-wave plate following *α* = *qφ*+*α*_0_, where *φ* is the azimuthal angle and *α*_0_ represents the initial axis angle. When an incidence illuminates an object, i.e. ${{E}_{{\bf in}}}(x,y) \propto {{T}_{{\bf obj}}}(x,y)$, and propagates through a 4*f* system with a FLC topological superstructure (i.e. FLC *q*-plate with *q* = 1/2) placed at the Fourier plane, the output optical field is:


(1) \begin{eqnarray*}
{{E}_{\bf{out}}}(x,y) = {{E}_{\bf{in}}}(x,y) \otimes h(x,y).
\end{eqnarray*}

The symbol $\otimes $ represents the convolution operation and *h*(*x, y*) denotes the Fourier transform of the transfer function *H*(*x, y*) and acts as the point-spread function (PSF). Here, under the specific condition of crossed polarizers, the transfer function can be expressed as:


(2) \begin{eqnarray*}
{H(x,y)} &=& \left[\! {\begin{array}{cc} {\cos \displaystyle\frac{{3\pi }}{8}}&{\sin \displaystyle\frac{{3\pi }}{8}} \end{array}} \right]
\left[ {\begin{array}{@{}*{2}{c}@{}} {\cos 2\alpha }&{\sin 2\alpha }\\ {\sin 2\alpha }&{ - \cos 2\alpha } \end{array}} \right]\\
&&\times \left[ {\begin{array}{@{}*{1}{c}@{}} {\cos \left( { - \frac{\pi }{8}} \right)}\\ {\sin \left( { - \frac{\pi }{8}} \right)} \end{array}} \right]\\
&=& \cos \left(\varphi + 2{{\alpha }_0} - \frac{\pi }{4} \right),
\end{eqnarray*}

where $[ _{{\cos ( { - \frac{\pi }{8}} )}}^{{\sin ( { - \frac{\pi }{8}} )}} ]$ and ${{[ {\! {\cos \frac{{3\pi }}{8}}\,\,\,\,{\sin \frac{{3\pi }}{8}}}\! ]}^T}$ represent the polarization state of the incident and output light, respectively.

According to Equation (2), the transfer function of the FLC device introduces the phase difference of π between the positive and negative spatial frequencies of the optical field at the direction parallel to –(2*α*_0_ – *π*/4) and functions similarly to 1D Hilbert transform filtering [8,31–33]. This direction can be described by a direction vector $\vec{u}$, $- (2{{\alpha }_0} - \frac{\pi }{4}) = \arccos \{ \frac{{\vec{u} \cdot (\vec{x}/| {\vec{x}} |)}}{{| {\vec{u}} |}}\} $, which is highly dependent on *α*_0_. Therefore, the convolution of *E*_in_ and the PSF causes the destructive interference in the uniform area and those edges are emphasized in the area with an intensity gradient along $\vec{u}$. In other words, real-time phase contrast imaging, equivalent to the functionality of the directional derivative $\frac{\partial }{{\partial \vec{u}}}$, is realized. Therefore, the edges along different orientations can be respectively enhanced by selecting distinguished *α*_0_.

Notably, the overall optical axis of the FLC topological superstructure can be successfully redistributed by the applied external electric field. When the electric field polarity alters, *α*_0_ will change by 2*θ* (from –*θ*/+*θ* to +*θ*/–*θ*), causing the selected edges to vary synchronously. As vividly exhibited in Fig. 1a, for *α*_0_ = +*θ* = +π/8 under the positive polarity of the external electric field, *H*(*x, y*) = cos(*φ*), which introduces the π phase shift at the left half of the filter plane, acting as the differential operation along the *x* direction. Thus, the vertical edges are selected and highly enhanced. Conversely, for the negative polarity (Fig. 1b), *α*_0_ = –*θ* = –π/8 and *H*(*x, y*) = cos(*φ* – π/2) = sin(*φ*), which results in a phase order change in the *y* direction and performs the differentiation along *y*. Therefore, the horizontal edges are selectively highlighted in the imaging process. In this design, the selected edges can dynamically turn from the horizontal/vertical edges to the vertical/horizontal edges, depending on the applied electric field.

### Reconfigurable topological hierarchical superstructures

**Figure 2.**
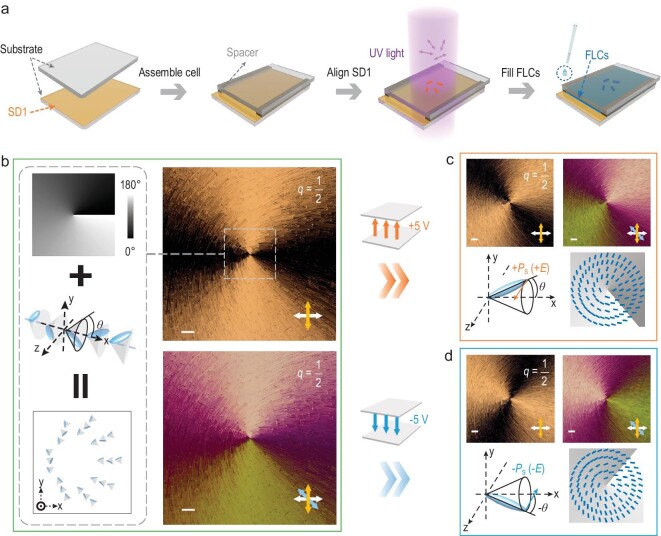
Fabrication process and optical micrographs of the FLC topological superstructures. (a) Fabrication process of the FLC sample. Double-ended arrows represent the local linear polarization distribution of the UV light. Yellow and blue short rods depict the SD1 orientation and the optical axis direction of the FLCs, respectively. (b–d) Expected distribution of the equivalent optical axis and optical micrographs of the fabricated FLC *q*-plate (*q* = 1/2) under crossed polarizers (b) without an external electric field, (c) under a positive electric field (+5 V) and (d) under a negative electric field (–5 V), respectively. White, yellow and blue double-ended arrows label the direction of the polarizer, analyser and sensitive tint plate, respectively. All scale bars are 100 μm. The relationship between the external electric field and the molecular rotation is illustrated. *θ* is the tilt angle, i.e. half of the cone angle of the FLC helical structure.

To fabricate an inhomogeneously aligned FLC sample with good quality, we introduced the photo-patterning technique and adopted asymmetric planar boundary conditions for the FLC material with helix pitch *P*_0_ = 245 nm, spontaneous polarization *P*_S_ = 110 nC/cm^2^ and tilt angle *θ* = 25°, whose phase transitions of isotropic state to chiral smectic A (SmA*) and SmA* to SmC* are at 82°C and 72°C, respectively. The fabrication process of the FLC topological hierarchical superstructure is schematically illustrated in Fig. 2a. Firstly, two bare indium tin oxide (ITO)-coated glass plates were prepared, with only one of them coated with the photo-alignment layer of sulfonic azo-dye SD1 (DIC, Japan). Next, certain spacers were used to form the sandwich-like configuration, with the required cell thickness of 1.5 μm. The thickness-to-pitch ratio here satisfies the condition of the FLC ESH mode. Then, the empty cell was exposed to ultraviolet (UV) light, which induced the SD1 molecules to be aligned perpendicularly to the illuminated UV polarization. Accordingly, the predesigned *q*-plate (*q* = 1/2) pattern (inset of Fig. 2b) was memorized in the SD1 layer through the multistep polarized exposure [34,35]. Following this, the FLC materials were filled into the photo-patterned cell with the temperature over the clearing point and gradually cooled from isotropic phase to SmC* phase at a rate of 1°C/min. In particular, the cooling rate was further decreased to 0.1°C/min near the ±2°C range of the phase transition points. This slow cooling process promotes FLCs to adequately self-organize with low dislocation density and constructs the topological hierarchical superstructures directed by the azimuthally variant boundary condition.

Figure 2b illustrates the expected optical axis distribution with azimuthal variation. As a typical chiral structure in FLCs, the unit directors are confined to the local tilt cone and oriented around the helix axis, which is also regarded as the equivalent local optical axis and is parallel to corresponding normal of local smectic layer. When a FLC phase is subjected to a particular planar boundary condition, the equivalent optical axis lies in the plane of the substrate and along the direction of alignment. In this case, the FLC device is formed by parallel smectic layers that are bent around the central topological defect point. Polarized micrographs on the right of Fig. 2b exhibit the fantastic texture of the fabricated FLC *q*-plate with *q* = 1/2 without applying an electric field. The space-variant intrinsic defect lines perpendicular to the smectic layers imply the topological chiral lamellar superstructures. Also, the continuous brightness changing indicates the optical axis distribution following *α* = *φ*/2, as expected. By inserting a full-wave plate of 530 nm at 45° to the crossed polarizers as a sensitive tint plate, an enhanced anisotropic texture is further obtained with distinct colors.

Owing to the coupling between the spontaneous polarization of FLCs and the external electric field, such chiral superstructures can be unwound by applying a direct-current voltage perpendicular to the helix axis. Figure 2c and d exhibits the optical micrographs of the FLC topological superstructures under the electric field with opposite polarities, showing the marvelous transformation of the optical axis (see corresponding dynamic change in Movie S1). The FLC molecules rotate to either side of the core surface in the *x*–*y* plane, depending on the reorientation of the spontaneous polarization. Therefore, the equivalent optical axis redistributes synchronously. The defect lines in all micrographs remain unchanged because the azimuthally variant lamellar structure is fixed. Just the overall optical axis rotates by +*θ*/–*θ* under a positive/negative electric field, which is clearly verified by the changing of the brightness and color distribution in the tint-plate-inserted micrographs.

### Dynamic light manipulation

**Figure 3.**
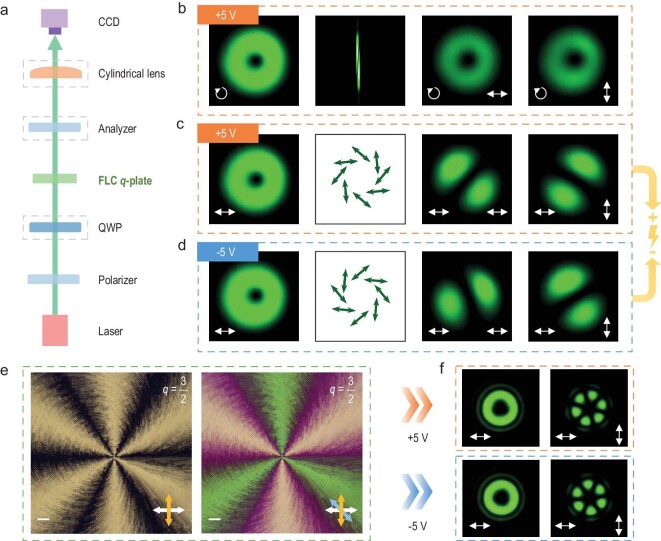
Optical set-up for characterizing the FLC *q*-plate and the generated vector beams under different external electric fields. (a) Optical set-up to generate and analyse the vector beams from the FLC *q*-plate. QWP, quarter-wave plate; CCD, charge-coupled device. (b–d) Intensity profiles and polarization distributions of the generated vector beams at 550 nm under different incident polarization and applied voltages: (b) LCP incidence under +5 V, (c) LP incidence under +5 V and (d) LP incidence under –5 V. The incident polarization and the direction of the analyser are labeled by arrows in the bottom left-hand and right-hand sides, respectively. The detection result of the topological charge of the optical vortex is also presented in (b) for LCP incidence. (e) Micrographs of the other *q* = 3/2 FLC *q*-plate. Left: the micrograph recorded under crossed polarizers, with white and yellow double-ended arrows labeling the polarizer and analyser, respectively. Right: the sensitive-tint-plate-inserted micrograph recorded under crossed polarizers, with white, yellow and blue double-ended arrows labeling the polarizer, analyser and tint plate, respectively. All scale bars are 100 μm. (f) Diffraction patterns with LP incidence under opposite electric field polarities, with the polarizer and the analyser labeled by arrows in the bottom left-hand and right-hand sides, respectively.

The capability of dynamic light manipulation via the FLC topological superstructure is investigated by using the optical set-up shown in Fig. 3a. For left circularly polarized (LCP) incidence at 550 nm, a donut-like intensity profile of the orthogonal-circularly polarized optical vortex is obtained under a positive external electric field (Fig. 3b). The polarization conversion efficiency, defined as the power ratio of the output circular polarization opposite to the incidence to the total transmission light, is measured at as 97% owing to the highly efficient pure phase modulation. After adding a rotatable analyser, the intensity decreases while the shape of the pattern remains unchanged. A cylindrical lens [17] is inserted to verify *q* = 1/2. Similar patterns can also be observed under a negative electric field (Fig. S1). Meanwhile, for the linearly polarized (LP) incidence (Fig. 3c and d), the polarization distribution of the vector beams [36,37] can also be revealed. When the polarity is positive, a typical donut-shaped profile becomes two radial lobes after transmitting the analyser. For the negative voltage, the polarization distribution is overall converted, as indicated by the ∼100° rotated lobe patterns. These verify an electrically switchable polarization distribution. Notably, FLC *q*-plates with larger topological charges and accordingly vector beams with denser polarization variations can also be realized. An example of *q* = 3/2 is demonstrated in Fig. 3e and f. The left panel of Fig. 3e exhibits its optical micrograph under crossed polarizers, in which a drastic azimuthal change in the brightness and defect lines are clearly observed. The right panel of Fig. 3e provides an enhanced anisotropic texture with an additional sensitive tint plate inserted. The distinct colors vividly demonstrate the azimuthally variant optical axis (Fig. S2). Correspondingly, the diffraction pattern under crossed polarizers, containing 4*q* radial lobes, converts to a nearly complementary pattern by alternating the electric field polarity (Fig. 3f and Fig. S3).

### Fast selective and broadband edge-enhanced imaging

**Figure 4.**
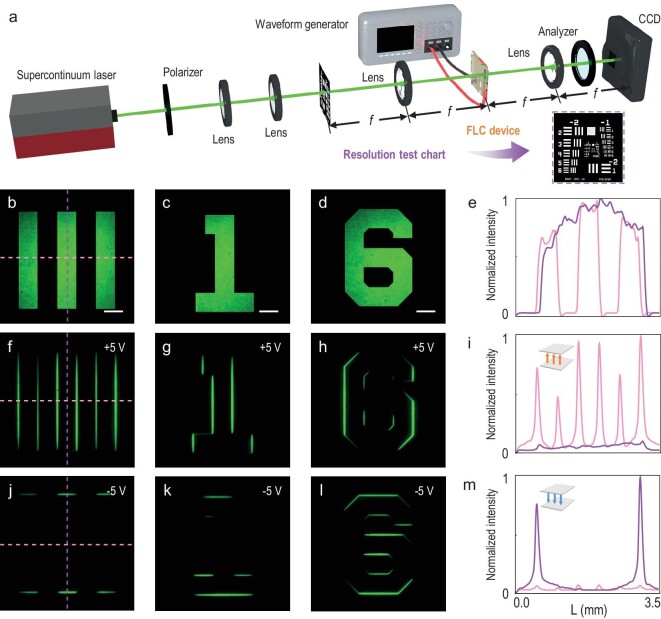
Dynamically switchable edge-enhanced imaging by the proposed FLC topological superstructures. (a) Experimental set-up of optical edge detection based on the FLC device. Inset: 1951 USAF resolution test chart. (b–d) The bright images without the FLC device. (f–h) The vertical edge images under +5 V. (j–l) The horizontal edge images under –5 V. (e), (i) and (m) are corresponding intensity analyses of the dotted lines marked in (b), (f) and (j), respectively. All scale bars are 500 μm.

Furthermore, we place the FLC device at the confocal plane of a 4*f* system (Fig. 4a). A Gaussian beam at 550 nm is adjusted to the linear polarization oriented at –22.5°. As the object to be imaged, a 1951 United States Air Force (USAF) resolution test chart is placed at the front focal plane and shapes the expanded incident beam. Vertical lines belonging to Element 1 of Group 0 on the chart, whose resolution is 1 mm per line pair, are chosen along with the number ‘1’ and the number ‘6’ from Group–1. The analyser is always set perpendicular to the polarizer and an external electric field is applied by using a waveform generator. Corresponding bright images without the FLC device are shown in Fig. 4b–e. By alternating the positive or negative voltage, the overall optical axis of the FLC topological superstructure reorientates consistently with the theoretical design, allowing the dynamic switch between two nearly orthogonal directions of edge detection. When the polarity of the external electric field is positive, the FLC *q*-plate with *q* = 1/2 performs as the optical differentiator in a nearly horizontal direction, selectively enhancing the vertical edges (Fig. 4f–i). Conversely, under the negative field, it detects the horizontal edges (Fig. 4j–m), working as the differentiator in a nearly vertical direction. Respective normalized intensity distributions (Fig. 4e, i and m) quantitatively verify the highly selective, high-contrast and high-quality edge detection results. We provide a dynamic switching of the optical edge detection in Movie S2 and more object imaging results in Fig. S4.

**Figure 5.**
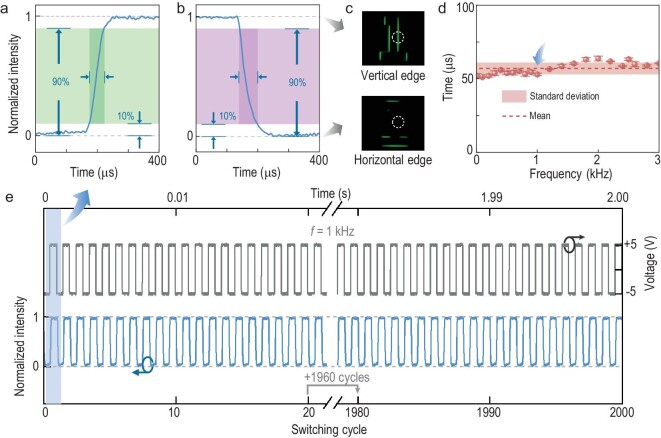
Fast response of the selective edge-enhanced imaging. Response time of (a) the horizontal edge detection state switching to the vertical edge state and (b) vertical to horizontal, with the respective edge detection results in (c). (d) Response time versus the frequency of the applied signal. The dashed line demonstrates the mean value of 57 µs within the frequency range of ≤3 kHz. The rectangular area illustrates the error analysis, with the standard deviation at 4.0 µs. (e) 2000 switching cycles at 10 Vpp and 1 kHz.

The exceptional electro-optical properties of ESH–FLCs allow an ultra-fast switching speed in the selective edge detection system. The switching time between the vertical and horizontal edge imaging state, which is defined as the duration of the intensity change between 10% and 90% (Fig. 5a and b), is obtained by measuring the relative intensity of the circled part shown in Fig. 5c. When applying the alternating-current square wave signal of 10 Vpp and 1 kHz, the switching time of the horizontal to vertical edges and the opposite process are measured as 53 and 60 µs, respectively. Such an electro-optical response is approximately two to three orders of magnitude faster than those of common LC devices. The switching time versus the frequency (≤3 kHz) of the applied signal is also investigated and a stable response of ∼57 µs with small fluctuations is presented in Fig. 5d. To further demonstrate the reliability and reversibility of the proposed FLC optical device, thousands of switching cycles are successfully verified, with 2000 cycles shown in Fig. 5e. The response curve maintains the stable wave shape without any obvious change during the whole process, vividly exhibiting its superior dynamic performance. Such a fast response may make this FLC device compatible with popular commercial high-speed complementary metal oxide semiconductor sensors or charge-coupled devices whose frame rates are usually at the range of 10^3^ to 10^4^ fps, contributing a smoother imaging display.

**Figure 6.**
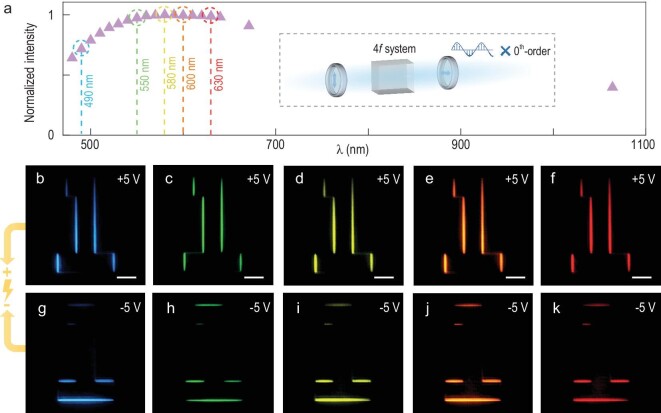
Broadband and fast selective edge detection. (a) Normalized intensity of the same detected edges at different wavelengths. Inset: working principle of the proposed broadband framework, where 0th-order light is filtered by the crossed polarizer. (b–f) The vertical edge-enhanced imaging under +5 V and (g–k) the horizontal edge-enhanced imaging under –5 V at 490, 550, 580, 600 and 630 nm, respectively. All scale bars are 500 μm.

It is important to note that our proposed system can wholly suppress the undesired bright imaging part, i.e. the 0th-order unchanged light component, by using the crossed polarizer and analyser. This allows ultra-broadband edge-enhanced imaging with the same fast selective advantage. The dependence of the normalized intensity of the same detected edges on the incident wavelength is depicted in Fig. 6a. Despite filtering the unmodulated light, the desired functionality remains impressive from the visible to near infrared spectrum. We choose working wavelengths of 490, 550, 580, 600 and 630 nm as five representative examples to demonstrate the broadband performance. Corresponding selective edge detection images under different electric field polarities are presented in Fig. 6b–k, respectively, indicating a polychromatic and efficient edge enhancement method with good electrical controllability.

## DISCUSSION

In summary, we propose an innovative strategy for dynamic optical edge detection based on an FLC topological superstructure. By photo-patterning chiral lamellar structure with azimuthal distribution, the FLC device can modulate nearly orthogonal vector beams by controlling the polarity of the applied external electric field. High-contrast and broadband edge-enhanced imaging is efficiently realized on demand. Horizontal and vertical edges are flexibly selected and switched with an ultra-fast response. The excellent reliability and reversibility of the dynamic edge imaging are vividly verified as well. Besides, edge detection along more directions can be conveniently achieved by just rotating the proposed FLC device. For instance, rotating by 22.5° successfully highlights the 45° or 135° edges, as demonstrated in Figs S5 and S6. The proposed high-speed FLC device provides a fast-switchable solution between orthogonal directional derivative operations, which is promising to work as the controllable processor in optical computing. In addition, the rapid edge selection might be compatible with commercial high-speed imagers, benefitting the fast acquisition and real-time exhibition of multidimensional light information. This work explores the potential of topological heliconical superstructures in the emerging frontiers of analog optical computing and discloses their unprecedented capabilities in the fields of machine vision.

## MATERIALS AND METHODS

### Materials

The sulfonic azo-dye SD1 (DIC, Japan) was dissolved in dimethylformamide at a concentration of 0.35 wt%. The FLC material (BEAM Co., USA) with helix pitch *P*_0_ = 245 nm, spontaneous polarization *P*_S_ = 110 nC/cm^2^ and tilt angle *θ* = 25° near ideal 22.5° was chosen, with a phase transition of isotropic → SmA* → SmC* at 82°C and 72°C, respectively. The thickness-to-pitch ratio in our experiment is ∼6, meeting the condition of the FLC ESH mode.

### Sample fabrication

Two bare ITO-coated glass substrates (1.5 × 2.0 cm^2^) were prepared. Only one of them was UV-Ozone cleaned, embedded with the photo-alignment layer of SD1 and cured at 100°C for 10 min sequentially. Certain spacers were adopted to assemble the two substrates and form the sandwich-like configuration with the required cell thickness of 1.5 μm (Fig. 2a). The predesigned *q*-plate pattern was memorized in the SD1 layer through multistep polarized exposure with the digital-micromirror-device-based photo-patterning system [34,35]. The FLC materials were filled into the photo-patterned cell at 85°C and then gradually cooled from isotropic phase to SmC* phase at a rate of 1°C/min. Notably, the cooling rate was further reduced to 0.1°C/min when approaching the phase transition points within the range of ±2°C.

### Characterizations

Temperature control was conducted by using a hot stage (LTS120, Linkam, UK). All micrographs were recorded by using a polarized optical microscope (Ci-POL, Nikon, Japan) with a crossed polarizer and analyser under the transmission mode. The intensity of light was detected by using a digital optical power meter (PM100D, Thorlabs, USA). The experiments in Figs 3, 4 and 6 utilized a supercontinuum fiber laser (SuperK EVO, NKT Photonics, Denmark) filtered at different wavelengths by using a multichannel acousto-optic tunable filter (SuperK SELECT, NKT Photonics, Denmark). The incident polarization was controlled by using a polarizer and a quarter-wave plate, and the transmitted light was optionally filtered via an analyser and another quarter-wave plate. The driving electric field was generated by using a waveform generator (33500B, Keysight Technologies, USA). The electrical response curve was measured by using a photodetector (PDA100A-EC, Thorlabs, USA) and an oscilloscope (MDO34, Tektronix, USA).

## Supplementary Material

nwae247_Supplemental_Files

## Data Availability

All data supporting this study and its findings are available within this published article and its Supplementary information files. Any other relevant data are available from the Corresponding authors upon request.
