# Supplementary material for: Fast selective edge-enhanced imaging with topological chiral lamellar superstructures
Source: Natl Sci Rev. 2024 Jul 17;11(11):nwae247. doi: 10.1093/nsr/nwae247 (PMC11493092; doi:10.1093/nsr/nwae247)
Supplement: nwae247_Supplemental_Files [file nwae247_supplemental_files.zip › Revised Supplementary Information_NSR_MS-2024-376.R1.pdf]

Supplementary Information for

Fast selective edge-enhanced imaging with topological chiral lamellar superstructures

Wen Chen[1]†, Dong Zhu[1]†, Si-Jia Liu[1], Yi-Heng Zhang[1], Lin Zhu[1], Chao-Yi Li[1], Shi-Jun Ge[1], Peng Chen[1]*, Wan-Long Zhang[2]*, Xiao-Cong Yuan[2]* and Yan-Qing Lu[1]*

[1]National Laboratory of Solid State Microstructures, Key Laboratory of Intelligent Optical Sensing and Manipulation, College of Engineering and Applied Sciences, and Collaborative Innovation Center of Advanced Microstructures, Nanjing University, Nanjing 210093, China.
[2]Nanophotonics Research Center, Institute of Microscale Optoelectronics, Shenzhen University, Shenzhen 518060, China.

*E-mail: chenpeng@nju.edu.cn, zwl@szu.edu.cn, xcyuan@szu.edu.cn, yqlu@nju.edu.cn
† These authors contributed equally to this work.

This Supplementary Information contains six Supplementary Figures, and two Supplementary Movies.

Supplementary Figures

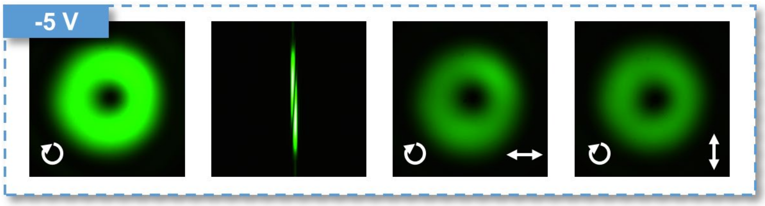

Figure S1. Diffraction patterns of the fabricated *q* = 1/2 FLC *q*-plate under LCP incidence and negative external electric field. Corresponding optical vortex detection result is also presented. The incident polarization and the direction of the analyzer are labeled by while arrows in the bottom lefthand and righthand side, respectively.

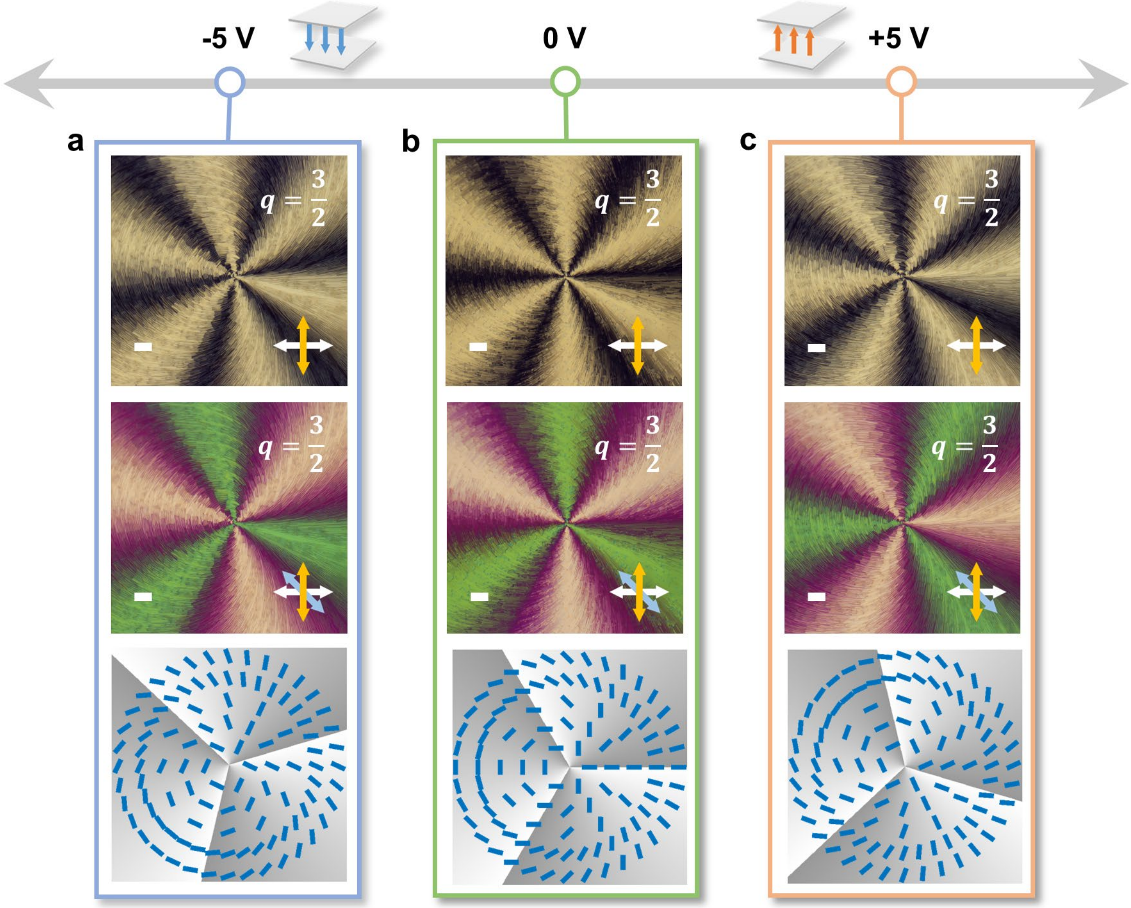

Figure S2. Polarized micrographs and the designed optical axis distribution of the *q* = 3/2 FLC *q*-plate at (**a**) -5 V, (**b**) 0 V, and (**c**) +5 V. All scale bars are 100 μm. White, yellow and blue double-ended arrows label the direction of polarizer, analyzer and sensitive tint plate, respectively. Blue short line illustrates the local equivalent optical axis direction, while the grayscale images lining at the bottom indicate the azimuthally-variant optical axis distribution.

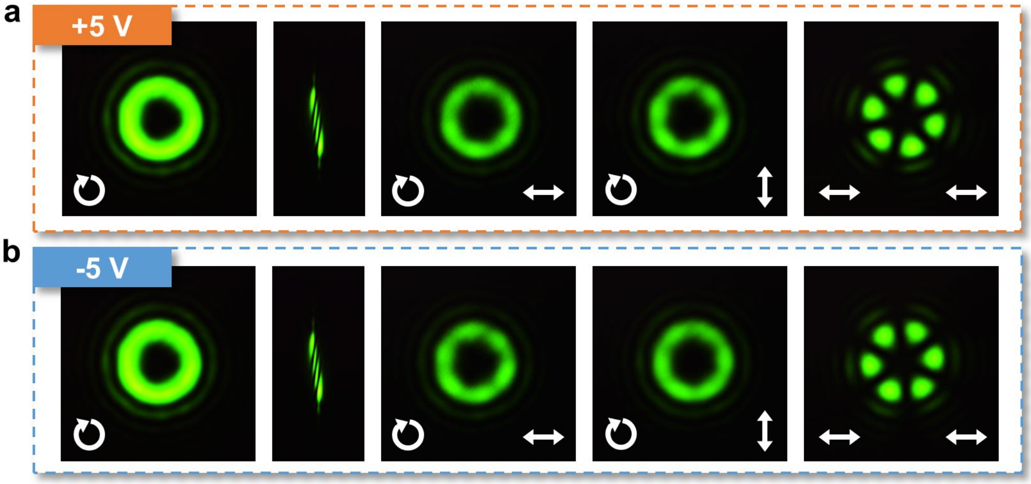

Figure S3. Diffraction patterns of the fabricated q = 3/2 FLC q-plate under opposite electric field polarities. Corresponding optical vortex detection results are respectively presented. The incident polarization and the direction of the analyzer are labeled by while arrows in the bottom lefthand and righthand side, respectively.

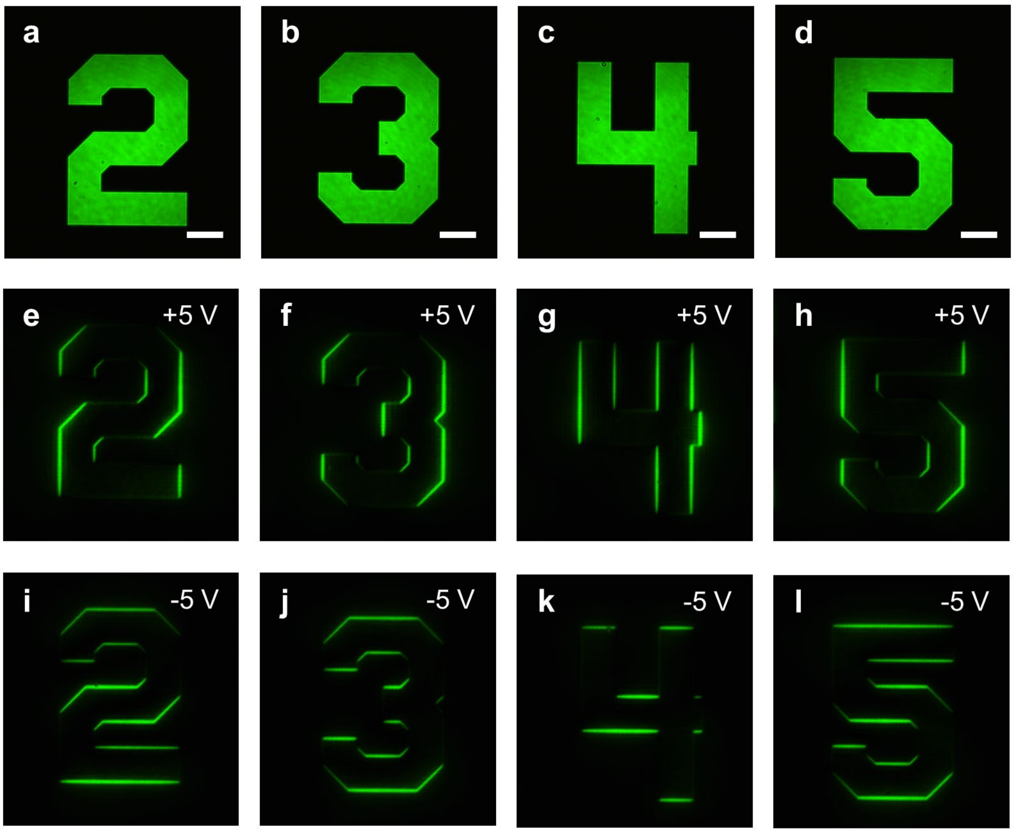

Figure S4. Selective edge-enhanced imaging for more imaging objects. The number '2', the number '3', the number '4', and the number '5' belonging to the group -1 on the 1951 USAF resolution test chart are chosen as imaging objects. (**a**)-(**d**) The bright images without the FLC device. (**e**)-(**h**) The vertical edge images under +5 V. (**i**)-(**l**) The horizontal edge images under -5 V. All scales are 500 μm.

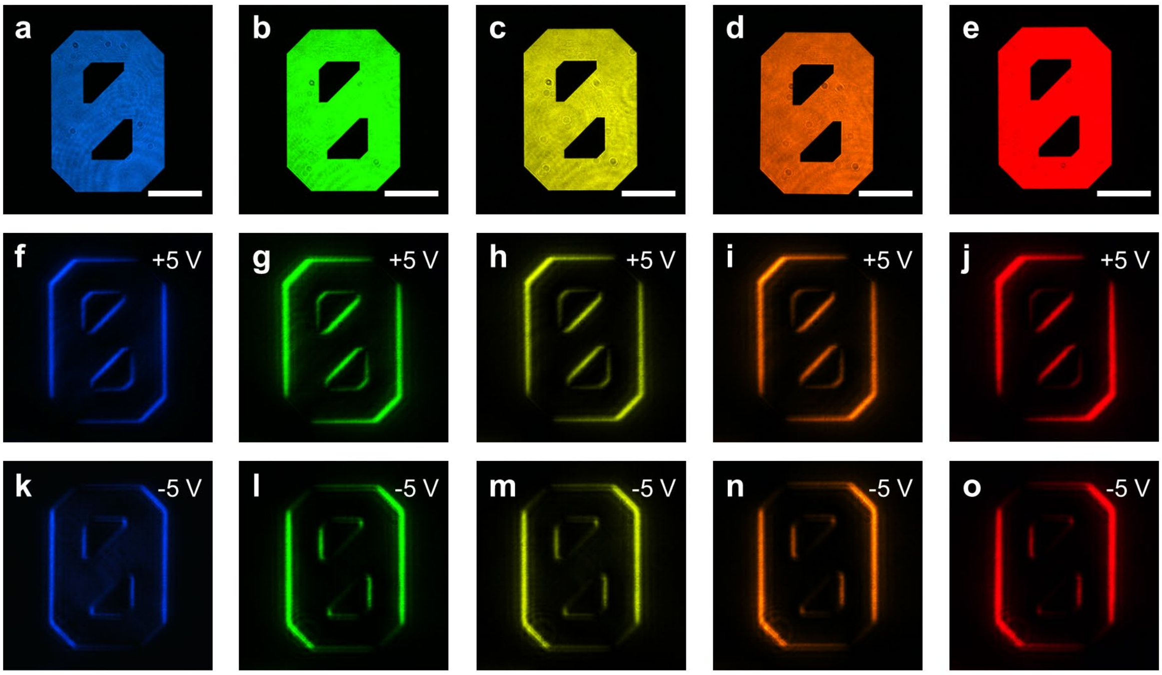

Figure S5. Broadband edge detection for the number '0' from the group 0 on the 1951 USAF resolution test chart. (a)-(e) The bright images without the FLC device, (f)-(j) the 45°-edge-enhanced imaging under +5 V, and (k)-(o) the 135°-edge-enhanced imaging under -5 V at 490 nm, 550 nm, 580 nm, 600 nm, and 630 nm, respectively. All scare bars are 500 µm.

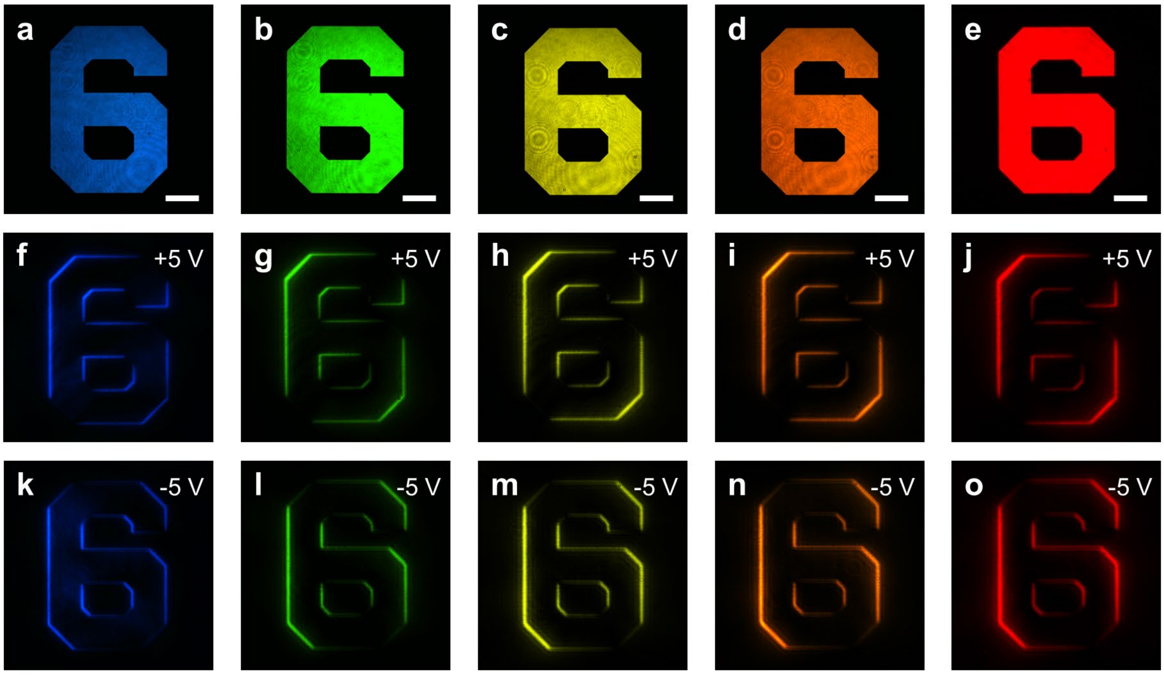

Figure S6. Broadband edge detection for the number '6' from the group -1 on the 1951 USAF resolution test chart. (a)-(e) The bright images without the FLC device, (f)-(j) the 45°-edge-enhanced imaging under +5 V, and (k)-(o) the 135°-edge-enhanced imaging under -5 V at 490 nm, 550 nm, 580 nm, 600 nm, and 630 nm, respectively. All scare bars are 500 µm.

Supplementary Movies

Movie S1. Dynamic switching of the micrograph under different electric field

In this movie, the polarized optical micrograph of the q = 1/2 FLC q-plate switches between two states under the external electric field with opposite polarities. When the positive voltage is applied, the micrograph is characterized as bright brushes. For the negative voltage, the equivalent optical axis is overall transformed, as reflected by the 100° rotated bright-brush patterns in the micrograph.

Movie S2. Dynamic switching of the optical edge detection

In this movie, the dynamic switching of two nearly orthogonal dimensions in the edge detection can be observed, verifying the highly selective and high-contrast detection results. When the polarity of the external electric field is positive, the proposed FLC optical device selectively enhances the vertical edge of the number '1'. On the contrary, in the case of negative voltage, it picks up the horizontal edge of the number '1'.
